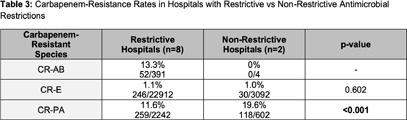# Mapping Microbial Resistance: Unveiling Regional Patterns through Atlanta’s Antibiogram Development

**DOI:** 10.1017/ash.2025.261

**Published:** 2025-09-24

**Authors:** Melat Endashaw, Trinh Vu, Sujit Suchindran, Edo McGee

**Affiliations:** 1 Emory University Hospital Midtown; 2 Emory University Hospital Midtown; 3 Emory University School of Medicine; 4 Philadelphia College of Osteopathic Medicine, School of Pharmacy

## Abstract

**Background:** Multidrug resistance remains one of the top global health threats and has been rising over recent decades, jeopardizing patient outcomes and increasing healthcare costs. This underscores an urgent need to design tools to optimize antibiotic prescribing to target these pathogens. Antibiograms are an essential antimicrobial stewardship tool used to provide guidance for empiric antimicrobial selection and information on local resistance. However, facility-level antibiograms are limited to individual institutions and do not reflect regional variations in resistance. Previous studies have demonstrated the feasibility and importance of generating regional antibiograms to better inform regional infection prevention and spearhead antimicrobial stewardship initiatives. Regional antibiograms also offer a valuable resource for community hospitals and health centers with lower pathogen prevalence and limited access to infectious diseases-trained personnel. This study aims to curate a regional antibiogram to analyze and understand antimicrobial susceptibility and resistance patterns of targeted pathogens across Metro Atlanta. **Methods:** This descriptive study aimed to evaluate antibiograms from multiple hospitals across the Atlanta metropolitan area. In September 2019, flagship hospitals of five different health-systems within metro Atlanta were surveyed using a questionnaire to collect information on basic facility and microbiology laboratory characteristics. Three health-systems responded, providing inpatient antibiogram data from the 2019 calendar year. These data were combined to create a single, cumulative antibiogram with 18 clinically relevant combinations of microorganisms and antibiotics. In total, data from 10 different hospitals were aggregated to create one regional antibiogram. **Results:** Data from 10 hospitals were combined to create one regional antibiogram with 18 organisms and 21 antibiotics. The overall prevalence of methicillin-resistant Staphylococcus aureus (MRSA) was 45.1% and vancomycin-resistant Enterococcus (VRE) was 15.8%, carbapenem-resistant Acinetobacter baumannii (CRAB) was 13.2%, carbapenem-resistant enterobacterales (CRE) was 1.1%, and carbapenem-resistant Pseudomonas aeruginosa (CR-PA) was 13.3%. Carbapenem resistance rates were compared between carbapenem-restrictive (n=4) and carbapenem-non-restrictive (n=2) hospitals. The prevalence of CR-PA was significantly higher in carbapenem-non-restrictive hospitals compared to carbapenem-restrictive hospitals, 19.6% vs 11.6% (p < 0 .001). **Conclusion:** The development of a regional cumulative antibiogram to capture resistance patterns of targeted pathogens across multiple health-systems in a large metropolitan area is feasible. Data from a regional antibiogram is useful in assessing susceptibilities and can serve as a valuable antimicrobial stewardship tool for institutions without access to their own specific antibiogram. Additionally, implementation of targeted stewardship policies, such as carbapenem restriction, demonstrates promise to potentially slow the development of resistant pathogens, thereby improving patient outcomes.

**Figure:**
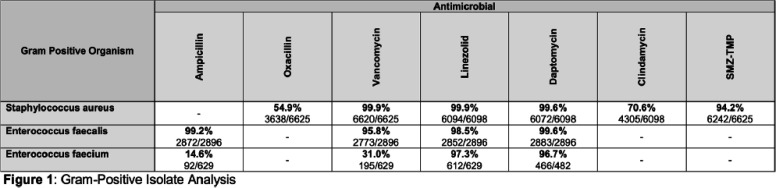

**Figure:**
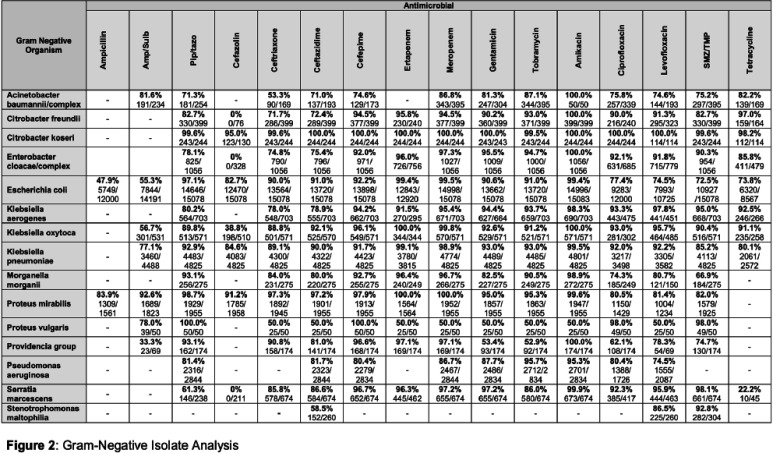

**Figure:**
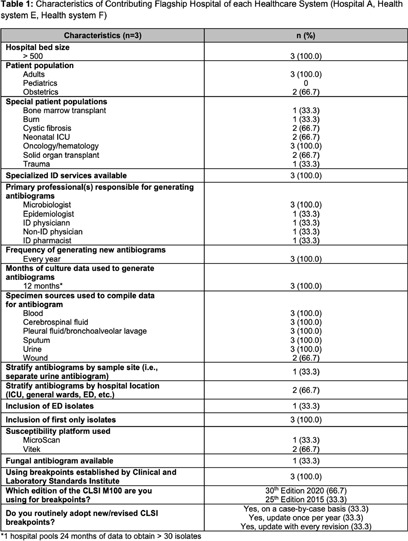

**Figure:**
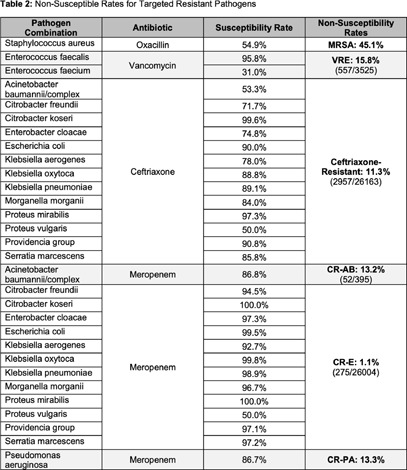

**Figure:**